# An Investigation of Situational and Dispositional Antecedents of Faking Intentions in Selection Interviews

**DOI:** 10.3389/fpsyg.2020.02034

**Published:** 2020-08-27

**Authors:** Benedikt Bill, Klaus G. Melchers, Anne-Kathrin Buehl, Sabine Wank

**Affiliations:** ^1^Institut für Psychologie und Pädagogik, Universität Ulm, Ulm, Germany; ^2^Carl Zeiss, Oberkochen, Germany

**Keywords:** selection interviews, impression management, faking, personality, honest impression management, competition, verification, warning

## Abstract

Applicants use faking in selection interviews to create a favorable impression and to increase their chances for a job offer. Theoretical models assume that such a behavior is influenced by situational and dispositional variables. However, previous research has mainly focused on dispositional variables whereas research about situational variables is sparse. To address this gap, we conducted three studies in which we examined how competition for a job and warning interviewees that information from their answers will be verified can influence faking intentions. Furthermore, we wanted to know whether these situational variables are able to explain additional variance in faking intentions beyond dispositional variables and whether there are interactions between situational and dispositional variables. In Study 1, we only found that high competition led to slightly higher faking intentions than low competition in a student sample. In Study 2, only a warning about the verification of applicants’ answers led to slightly lower faking intentions compared to no warning concerning verification in a working sample. Furthermore, faking intentions were lower in Study 2 than in the student sample in Study 1. In Study 3, we found no impact of our situational variables in a combined sample of students and non-students. We only found slightly higher honest impression management intentions in the high competition and the verification warning condition. We also found hardly any support for interaction effects between the situational and dispositional variables. Furthermore, the situational variables did not explain additional variance beyond the dispositional variables in any of the three studies. Possible reasons for the non-significant or small effect sizes for the situational variables can be found in a qualitative analysis of answers to an open-ended question in Study 3. However, we found that Honesty-humility und all facets of the Dark Triad were related to faking intentions. These results indicate that dispositional variables in particular have an impact on faking intentions.

## Introduction

Most applicants try to put their best foot forward in selection interviews to increase their chances to get a job offer. In order to create a favorable impression, they may use honest impression management to stress their true qualifications but also deceptive impression management (i.e., faking, Levashina and Campion, 2006). Both, honest and deceptive impression management are very prevalent and previous studies found that up to 99% of the applicants use corresponding impression management tactics in selection interviews (Levashina and Campion, 2007; Bourdage et al., 2018).

Even though different models assume that situational as well as dispositional factors influence the occurrence and extent of impression management in interviews (Levashina and Campion, 2006; Marcus, 2009; Roulin et al., 2016), a recent review mainly found studies related to dispositional factors (Melchers et al., 2020). Therefore, the first aim of the present paper was to examine the impact of two relevant situational factors on impression management (IM). To evaluate whether these situational factors can explain variance in IM beyond the effects of dispositional factors and whether there are interactions between situational and dispositional variables, we additionally collected information on personality antecedents. Furthermore, until recently, previous research only concentrated on deceptive IM (i.e., faking). However, applicants might also use honest IM to create a positive image in an interview (Levashina and Campion, 2006). Therefore, a second aim was to examine the effects of situational and dispositional factors on honest IM.

Altogether, we conducted three vignette studies to examine the impact of the situational factors, competition and warnings concerning verification of given answers, on the intention to show IM. Additionally, we measured the Big Five, the Dark Triad, and Honesty-humility to evaluate their effects. In Study 1, we examined a student sample. To evaluate the generalizability of our results to employed individuals, we conducted Study 2. To investigate the reasons for the differences that we found between Studies 1 and 2, we then conducted Study 3 in which we considered the participants’ background (students vs. non-students) as a quasi-experimental factor.

## Background

Deceptive IM is also called faking by many authors so that we use these two labels synonymously throughout the present paper. In the interview context, it is defined as “conscious distortions of answers to the interview questions in order to obtain a better score on the interview and/or otherwise create favorable perceptions” (Levashina and Campion, 2007, p. 1639). It contains the following subfacets that refer to different kinds of dishonest behavior (cf. Levashina and Campion, 2007): Image protection (hiding negative job-related information), ingratiation (making dishonest compliments to the interviewer/organization and adapting responses to the interviewer’s opinion), slight and extensive image creation (exaggerating or inventing of job-related abilities or skills). In contrast, applicants can also use honest IM to present themselves as good as possible without being untruthful (Levashina and Campion, 2006). Honest IM contains the subfacets honest self-promotion (emphasizing actually existing job-related competences), honest ingratiation (talking about common values and opinions), and honest defensive IM (giving honest explanations for past failures and resulting learning experiences) (Bourdage et al., 2018).

Levashina and Campion (2006) assumed that faking in selection interviews depends on interviewees’ capacity, willingness, and opportunity to fake. Interviewees with a high capacity to fake possess capabilities to fake effectively. These capabilities include oral skills, social skills, cognitive ability, and the knowledge about job roles and the constructs being measured. Willingness to fake represents interviewees’ internal tendency to distort their answers. It is influenced by personality but also by factors that affect interviewees’ motivation to fake such as the probability of getting caught, unfair treatment by the organization, interview coaching, and/or realistic job preview sessions. Finally, the opportunity to fake is defined by situational variables such as the interview type and format that can enable or hinder applicants’ faking.

In contrast to interview faking, there is no existing model for honest IM in selection interviews. However, Bourdage et al. (2018) assumed that Levashina and Campion’s (2006) interview faking model can also be used to understand antecedents of honest IM. Accordingly, honest IM should also depend on interviewees’ capacity, willingness, and opportunity to engage in honest IM. However, Bourdage et al. (2018) found that faking and honest IM differed in their antecedents. In their studies, applicants showed more honest IM when they were motivated to do well, interview questions were less difficult, and they were high on Conscientiousness. In contrast, applicants showed more faking when they were less motivated to do well, interview questions were more difficult, and they were high on Competitive Worldviews as well as low on Conscientiousness and Honesty-humility. Furthermore, even though honest IM and faking were positively correlated in these studies, they were nevertheless distinct constructs.

## Situational Antecedents

Even though various previous studies investigated antecedents of interview faking, the vast majority of these studies focused on dispositional antecedents (cf. Melchers et al., 2020; also see below) but only very few focused on situational antecedents of faking and honest IM. Previous research revealed that probing (i.e., follow-up questions), a higher difficulty of the interview, and less sophisticated questions led to more faking (Levashina and Campion, 2007; Bourdage et al., 2018). Furthermore, honest IM was positively associated with a longer interview duration as well as with less sophisticated questions and negatively with the difficulty of the interview (Bourdage et al., 2018). However, there is very little research on competition and warnings, two of the most obvious situational antecedents. With regard to the first of these antecedents, Ellingson (2011) stated that “people fake only when they need to fake” (p. 19). Obviously, when applicants know or assume that they have to compete with many other applicants then this influences their perceived necessity to engage in faking. In line with this idea, different authors found that competitive worldviews are related to faking in interviews (Roulin and Krings, 2016; Roulin and Bourdage, 2017). With regard to warnings as another situational antecedent, using warnings to engage in faking is an obvious measure to prevent applicants from actual faking. Furthermore, it turned out that warnings are suitable for reducing faking in personality tests (McFarland, 2003) and there is also evidence that some warnings can reduce faking in interviews (Law et al., 2016). However, there are hardly any other possibilities for organizations to reduce faking in interviews without changing the interview itself. Therefore, more attention should be paid examining effects of warnings.

According to the dynamic model of applicant faking by Roulin et al. (2016), perceived competition has a direct impact on the motivation to fake and thus on actual faking. According to Roulin et al. (2016), this perceived competition is, for example, determined by the number of other applicants with equal or better qualifications. Thus, faking is described as an adaptive reaction to assert oneself against competitors. Snell et al. (1999) also assumed that a low selection ratio could lead to a higher motivation to fake in order to outperform qualified competitors.

So far, there are only a few results concerning the impact of competition in selection interviews on faking and these turned out inconsistently. In a vignette study by Buehl and Melchers (2018), participants in a high competition condition did not differ in their faking intentions from those in a low competition condition. In contrast, another vignette study by Ho et al. (2019) with a larger sample found a positive relation between perceived competition and faking intentions. Additionally, they found that perceived competition mediated the relation between the selection ratio and faking intentions. Given the larger power of the latter study and also given the theoretical assumptions stressing the role of competition for faking, we suggest:

Hypothesis 1: High competition leads to higher faking intentions than low competition.

Since faking and honest IM are positively related (Bourdage et al., 2018) and applicants probably also want to present their actual competences and qualifications in a highly competitive situation as good as possible, we also propose:

Hypothesis 2: High competition leads to higher honest impression management intentions than low competition.

According to Levashina and Campion’s (2006) faking model, applicants’ willingness to fake is higher if the probability of getting caught is low. Consequently, this model assumes that applicants should be less motivated to engage in faking if their answers are verified after the interview. Likewise, in their dynamic model of applicant faking, Roulin et al. (2016) proposed that applicants perceive less opportunity to fake when organizations invest in measures that increase the risk of being caught. This in turn should affect their faking behavior.

Previous research with personality tests has shown that an identification warning and/or a warning about consequences of faking leads to lower test scores (Dwight and Donovan, 2003). Similarly, warnings against faking had a direct negative impact on the intention to fake and on faking behavior in such tests (McFarland and Ryan, 2006). However, in the interview domain, only the study by Law et al. (2016) examined the impact of warnings on faking behavior. It found that people who received an identification warning compared to those who received no warning reported less faking behavior after the interview. In such a warning, participants were told that they would be asked questions in the interview that are suitable to find out whether their answers are true or not. In contrast to the effects of an identification warning, a moral warning stressing that being honest is the right thing and fair, and a combined warning consisting of an identification warning and an announcement of positive consequences for honest responding had no effect on faking behavior.

A problem with identification warnings is that they are untrue themselves because previous research has shown that interviewers are hardly able to detect faking in interviews (Roulin et al., 2014, 2015). Other attempts to detect faking, like an application of a criterion-based content analysis, are still too inaccurate to use them in practice (Roulin and Powell, 2018). Therefore, other approaches are needed to verify interviewees answers without being untruthful.

An alternative to a warning that faking can be detected during the interview would be to inform applicants that they have to name suitable references and that these references might be contacted to verify given answers. This requires interviewees to provide the interviewer with contact information of persons from their professional past in order to verify their answers from the interview. To our knowledge, no evidence concerning the effects of verification warnings is available. However, given that this kind of verification warning seems very similar to an identification warning in its consequence and also given previous results about effects of warnings in general, we suggest:

Hypothesis 3: A warning that answers from the interview will be verified leads to lower faking intentions than no warning about any verification.

## Dispositional Antecedents

As mentioned before, there is considerably more research on dispositional antecedents of interview faking compared to situational antecedents (cf. Melchers et al., 2020). In the context of the present study, we are particularly interested in dispositional variables such as the Big Five, Honesty-humility, and the Dark Triad to also assess whether situational variables have an impact on faking intentions beyond dispositional variables and whether there are interactions between situational and dispositional variables. Additionally, we are interested in relationships between dispositional variables and honest IM because there is also not much research on corresponding dispositional antecedents yet.

In their faking model, Levashina and Campion (2006) proposed that applicants’ willingness to fake is positively related to Extraversion. Additionally, extraverts assume that they have better lie telling abilities relative to others and are more successful than introverts in doing so (Kashy and DePaulo, 1996; Elaad and Reizer, 2015). However, previous studies found no consistent results concerning the relationship between faking and Extraversion (e.g., Buehl and Melchers, 2017; Roulin and Bourdage, 2017; Bourdage et al., 2018). Therefore, we pose the following research question:

Research Question 1: Is there a positive correlation between Extraversion and the intention to fake?

In contrast to this, people high on Conscientiousness should be less willing to fake (Levashina and Campion, 2006). In line with this, McFarland and Ryan (2000) found that higher Conscientiousness was associated with less faking in personality tests. This effect was also found in selection interviews where Conscientiousness was negatively associated with faking (Roulin and Bourdage, 2017; Bourdage et al., 2018). Therefore, we suggest:

Hypothesis 4: There is a negative correlation between Conscientiousness and the intention to fake.

According to Levashina and Campion’s (2006) faking model, Neuroticism is positively related to the willingness to fake. In line with this, positive correlations were found between Neuroticism and faking in personality tests (e.g., McFarland and Ryan, 2000) and selection interviews (Buehl and Melchers, 2017; Bourdage et al., 2018). Accordingly, we suggest:

Hypothesis 5: There is a positive correlation between Neuroticism and the intention to fake.

According to Levashina and Campion (2006), people high on Agreeableness should be less willing to fake. Agreeableness is also associated with lower self-rated lie telling abilities (Elaad and Reizer, 2015). Surprisingly, however, previous studies often found no significant negative correlation between faking and Agreeableness (Buehl and Melchers, 2017; Roulin and Bourdage, 2017; Bourdage et al., 2018). Therefore, we pose the following research question:

Research Question 2: Is there a negative correlation between Agreeableness and the intention to fake?

Honesty-humility is one of the six dimensions of the HEXACO model (Ashton and Lee, 2008). It covers aspects such as sincerity, fairness, modesty, and greed avoidance (Ashton and Lee, 2008). Accordingly, the definition of Honesty-humility is already in contradiction to faking. Furthermore, in their model, Levashina and Campion (2006) already assumed that the willingness to fake is negatively related to integrity, which is relatively similar to Honesty-humility. In line with this, several studies found negative correlations between Honesty-humility and faking in selection interviews (Law et al., 2016; Buehl and Melchers, 2017; Roulin and Bourdage, 2017; Bourdage et al., 2018; Ho et al., 2019). Therefore, we posit:

Hypothesis 6: There is a negative correlation between Honesty-humility and the intention to fake.

There is only one study that considered the relation between Honesty-humility and honest IM. Bourdage et al. (2018) asked whether the Humility component stands in contradiction to honest IM but they found no significant correlation between these variables. However, more research is needed to clarify this possible relation. Therefore, we offer the following research question:

Research Question 3: Is there a negative correlation between Honesty-humility and the intention to show honest impression management?

The Dark Triad of personality consists of Psychopathy, Machiavellianism, and Narcissism. People high on these traits have a malevolent character and a tendency to be emotionally cold and duplicitous (Paulhus and Williams, 2002). Therefore, it is not surprising that Levashina and Campion (2006) assumed and found a positive correlation between Machiavellianism and the willingness to fake. In addition, more recent studies found positive correlations between all facets of the Dark Triad and faking intentions (Roulin and Krings, 2016) as well as actual faking behavior (Roulin and Bourdage, 2017). Therefore, we predict:

Hypothesis 7: There is a positive correlation between the Dark Triad and the intention to fake.

There is only a single study that investigated the relationship between the Dark Triad and honest IM. In that study, Roulin and Bourdage (2017) found no significant correlation between honest IM tactics and the traits of the Dark Triad. However, given the limited available evidence, there is more research needed in order to provide more clarity. Therefore, we pose the following research question:

Research Question 4: Is there a correlation between the Dark Triad and the intention to show honest impression management?

## Interplay of Situational and Dispositional Factors

As mentioned before, different faking models assume that dispositional as well as situational variables influence the occurrence and the amount of faking (Levashina and Campion, 2006; Roulin et al., 2016). The research reviewed above has shown that both are related to faking behavior or faking intentions when considered individually. However, a recent review of interview faking (Melchers et al., 2020) suggests that the few results concerning situational factors are relatively inconsistent. Furthermore, research that evaluates whether situational variables can indeed account for variance in faking intentions beyond the effects of personality antecedents is missing even though conceptually, both groups of antecedents should account for unique amounts of variance. But based on different faking models, we posit:

Hypothesis 8: Situational variables explain additional variance in faking intentions beyond dispositional variables.

In addition to the main effects of situational and dispositional variables on faking, different models also assume interactions between these two kinds of variables. Concerning such interaction effects, we would like to refer to two models that differ concerning whether situational or dispositional variables function as the moderator but that agree that interactions between both types of variables can be expected. First, in their faking model, McFarland and Ryan (2000) proposed that personality affects beliefs toward faking and thereby faking intentions. Furthermore, according to this model, the relation between beliefs toward faking and faking intentions is moderated by situational variables like verification warnings. Accordingly, verification warnings should weaken the relationship between dispositional variables and faking intentions because of the risk that one might get caught. Second, in contrast to McFarland and Ryan (2000), Roulin et al. (2016) assumed that attitudes toward faking moderate the relationship between perceived competition and the motivation to fake. These attitudes are influenced by applicants’ personality. Therefore, for applicants high on dark personality traits or low on integrity and honesty the effect of competition on faking should be stronger than for applicants who are low on dark personality traits or high on Integrity. Thus, even though the two models differ on whether they view dispositional or situational variables as the moderator both agree that they expect interactions between the two types of variables. Therefore, we want to test the following hypothesis:

Hypothesis 9: There are significant interactions between situational and dispositional variables with regard to the intention to fake.

## Overview of the Present Studies

In three studies, we evaluated how competition and a verification warning affect faking intentions. In addition, we measured the Big Five and Honesty-humility in Studies 1 and 2. These two studies focused on main effects of situational and dispositional variables. In Study 3, we collected data from a larger sample so that we had more statistical power to investigate possible interactions between situational and dispositional variables. Furthermore, because of the results in Studies 1 and 2, we replaced the Big Five with the Dark Triad in Study 3. Finally, we also measured the intention to show honest IM in Study 3 and added an open-ended question to assess possible reasons for showing (or not showing) faking.

## Study 1

### Methods

#### Participants

Hundred-twenty-seven undergraduate psychology students from Ulm university completed an online survey to partially fulfill a course requirement. They were recruited via an online platform. Additionally, we used flyers and mailing lists to gain more attention.

We excluded 30 participants, because they answered more than one content-related question incorrectly (see below), and one participant, because she was less than 18 years old. Thus, the final sample size was 96 (87 females, 8 males, 1 no specification). Participants’ mean age was 20.91 (*SD* = 2.42) and most participants already had prior interview experience (89.4%), *M* = 3.19 previous interviews (*SD* = 2.94).

#### Procedure

We used a 2 × 2 between-subjects design (high vs. low competition and verification warning vs. no warning) for our experiment, which was administered via an online survey. The participants first completed an informed consent form. Then, the different personality scales were presented. Following this, participants had to read one of four vignettes in which they were told to imagine that they were invited to a selection interview for a Master’s program in psychology.^1^ After they had read their respective vignette, they had to answer content-related questions and manipulation check questions. Then they were asked about their faking intentions in this situation. Finally, we asked them about demographic information.

#### Vignettes

The vignettes (which can be found here: https://osf.io/4b6ex) asked participants to imagine that they had almost completed their Bachelor’s degree and had applied for admission to an attractive Master’s program. Competition was manipulated with two different descriptions. In the high competition condition, participants were told that the media often reported that far too few places were available for Master’s programs in psychology and that even applicants with a markedly better grade point average did not get a place. Additionally, we described the selection ratio at the specific university as very low (70 places for 700 applicants). In contrast, participants in the low competition condition were told that the media reported that enough places for Master’s programs in psychology were available and that even applicants with a lower grade point average got a place and the selection ratio was described as high (70 places for 100 applicants).

To manipulate verification, participants in the verification warning condition were told to imagine that they had to send back information on potential references (e.g., instructors from their Bachelor’s program) and that the university would contact these references to verify their answers from the interview. Participants were told that applicants would be excluded from the application process, if they provided untrue information. In the no warning condition, no information about attempts to verify interviewees’ answers were given.

#### Measures

Unless stated otherwise, all items from the following measures in this and the subsequent studies had to be answered on 5-point scales from 1 = *I do not agree at all* to 5 = *I fully agree*.

##### Big Five

We measured the Big Five with a short version of the Big Five Inventory (BFI-K) by Rammstedt and John (2005). It captures the dimensions Extraversion, Agreeableness, Conscientiousness, and Neuroticism with 4 items each. For the sake of completeness we also included Openness with 5 items. Coefficient alpha ranged from 0.66 to 0.86.

##### Honesty-humility

Honesty-humility was measured with the corresponding scale from the German version (Moshagen et al., 2014) of the HEXACO-60 by Ashton and Lee (2009). It contains 10 items (alpha = 0.73) such as “I would never accept a bribe, even if it were very large.”

##### Faking intentions

The intention to fake was measured with an 11-item version (alpha = 0.84) of Levashina and Campion’s (2007) interview faking behavior scale that was initially used by Ingold et al. (2015) to measure faking behavior in an actual interview. This scale contains one item for each of the eleven subscales of the Levashina and Campion scale (e.g., “I would present other people’s experiences or achievements as my own.” or “I would invent something to give better interview answers.”). All items were adapted to measure behavioral intentions and had to be rated on 5-point scales from 1 = *to no extend* to 5 = *to a very great extent*.

##### Manipulation check

As a manipulation check, participants had to answer two items (alpha = 0.96) concerning perceived competition (e.g., “The competition for a place in the Master program is quite high.”) and three items (alpha = 0.79) concerning perceived verification (e.g., “The university will verify my information from the interview.”).

##### Content-related questions

To verify that participants had read their vignettes carefully, they had to answer five content-related questions (e.g., “I have received an invitation for a selection interview at a university.”) to be *true* or *not true*.

### Results

Correlations and descriptive information can be found in Table 1. To check whether our experimental manipulations worked as intended, we computed two separate *t*-tests with the independent variables competition and verification warning with the corresponding dependent variables perceived competition and perceived verification from the manipulation check. We found higher perceived competition in the high competition condition (*M* = 4.79, *SD* = 0.35) than in the low competition condition (*M* = 2.48, *SD* = 0.92), *t*(55.03) = 15.87, *p* < 0.01, *d* = 3.32. Similarly, we found higher perceived verification in the verification warning condition (*M* = 4.32, *SD* = 0.59) than in the no warning condition (*M* = 2.95, *SD* = 0.98), *t*(54.43) = 7.74, *p* < 0.01, *d* = 1.69.

**TABLE 1:** Correlations, means, and standard deviations among all study variables in Study 1.

Variable	*M*	*SD*	1	2	3	4	5	6	7	8	9	10	11
(1) Sex	0.90	0.31	−										
(2) Age	20.89	2.46	–0.17	−									
(3) Extraversion	3.55	0.91	0.36**	–0.16	0.86								
(4) Conscientiousness	3.67	0.75	0.10	–0.08	0.14	0.74							
(5) Neuroticism	3.15	0.91	0.04	–0.03	−0.30**	0.03	0.83						
(6) Agreeableness	3.24	0.72	0.07	–0.12	0.17	0.08	–0.15	0.66					
(7) Openness to experience	3.95	0.73	–0.14	0.21*	0.08	0.10	0.16	0.11	0.75				
(8) Honesty-humility	3.50	0.58	0.25*	0.01	0.08	0.10	–0.07	0.16	–0.00	0.73			
(9) Competition	0.54	0.50	0.09	–0.05	0.06	–0.07	0.08	0.06	0.00	–0.18	−		
(10) Verification warning	0.57	0.50	–0.01	–0.10	0.09	0.06	0.05	0.03	–0.08	–0.04	–0.08	−	
(11) Faking intentions	2.84	0.56	–0.01	0.02	0.06	–0.11	0.14	–0.06	–0.04	−0.42**	0.27**	0.02	0.84

N = 126. Sex was coded as 0 = male, 1 = female. Values in the diagonal show coefficient alpha for the different variables. *p < 0.05, **p < 0.01.

To examine the impact of the situational variables on faking intentions, we conducted a 2 × 2 ANOVA with the independent variables competition and verification warning. In line with Hypothesis 1, we found a significant main effect for competition, *F*(3,92) = 5.73, *p* = 0.02, η^2^ = 0.059 (cf. Table 2 for means and *SD*s). However, in contrast to Hypothesis 3, the main effect for verification warning was non-significant, *F*(3,92) = 0.00, *p* = 0.95, η^2^ = 0.000. Finally, the Competition × Verification interaction was also non-significant, *F*(3,92) = 1.42, *p* = 0.24, η^2^ = 0.015.

**TABLE 2:** Means and standard deviations for the dependent variable faking intentions in the different experimental groups in Studies 1, 2, and 3 and for honest IM intentions in Study 3.

Variable	Competition low		Competition high	
	No warning	Verification warning	No warning	Verification warning
	*M* (*SD*)	*M* (*SD*)	*M* (*SD*)	*M* (*SD*)
**Study 1:**				
Faking intentions	2.76 (0.87)	2.61 (0.58)	2.90 (0.41)	3.04 (0.47)
**Study 2:**				
Faking intentions	2.49 (0.56)	2.31 (0.46)	2.51 (0.60)	2.19 (0.64)
**Study 3 students:**				
Faking intentions	2.34 (0.63)	2.27 (0.52)	2.36 (0.62)	2.26 (0.59)
Honest IM intentions	3.71 (0.47)	3.83 (0.50)	3.84 (0.42)	3.85 (0.63)
**Study 3 non-students:**				
Faking intentions	2.11 (0.58)	2.05 (0.58)	2.14 (0.54)	2.06 (0.54)
Honest IM intentions	3.66 (0.45)	3.74 (0.49)	3.80 (0.46)	3.88 (0.51)

*IM = impression management.*

Next, we examined the correlations between the dispositional variables and faking intentions. In line with Hypothesis 6, there was a significant negative correlation between Honesty-humility and faking intentions, *r* = −0.42, *p* < 0.01. In contrast, no support was found for Hypotheses 4 and 5, given that the corresponding correlations for Conscientiousness, *r* = −0.11, *p* = 0.31, and Neuroticism, *r* = 0.14, *p* = 0.18, were non-significant. Concerning Research Questions 1 and 2, correlations between Extraversion, *r* = 0.06, *p* = 0.54, and Agreeableness, *r* = −0.06, *p* = 0.57, on one side and faking intentions on the other side were also non-significant. For the sake of completeness, we also determined the correlation for Openness, which was also non-significant, *r* = −0.04, *p* = 0.74. Finally, given that there is evidence that sex is related to interview faking (Melchers et al., 2020) and also to personality, we calculated partial correlations between faking intentions and the personality variables for this and the following studies. The largest difference across all studies was Δ*r* = 0.037. Moreover, no further significant correlations were found after controlling for sex.

To evaluate whether the situational variables can explain additional variance in faking intentions beyond the dispositional variables, we conducted hierarchical multiple regressions (cf. Table 3). We added the dispositional variables Extraversion, Conscientiousness, Neuroticism, Agreeableness as well as Honesty-humility in Step 1 and the situational variables competition and verification warning in Step 2. Step 1 accounted for a significant amount of variance (*R*^2^ = 0.22), but Honesty-humility was the only dispositional predictor that was significant. Furthermore, in contrast to Hypothesis 8, there was no significant change in the amount of explained variance from Step 1 to Step 2 and the situational predictors competition and verification were both non-significant.

**TABLE 3:** Hierarchical regression analyses of predictors of faking intentions in Studies 1, 2, and 3 and honest IM intentions in Study 3.

Step	Predictor	β	*R*^2^	Δ*R*^2^
**Study 1 (*N* = 96): faking intentions**				
1	Extraversion	0.16	0.22**	0.22**
	Conscientiousness	–0.09		
	Neuroticism	0.16		
	Agreeableness	0.01		
	Honesty-humility	−0.42**		
2	Competition	0.18	0.25**	0.03
	Verification	–0.03		
**Study 2 (*N* = 114): faking intentions**				
1	Extraversion	–0.04	0.25**	0.25**
	Conscientiousness	–0.15		
	Neuroticism	0.04		
	Agreeableness	0.00		
	Honesty-humility	−0.44**		
2	Competition	–0.04	0.28**	0.03
	Verification	–0.16		
**Study 3 (*N* = 711): faking intentions**				
1	Honesty-humility	−0.30**	0.26**	0.26**
	Machiavellianism	0.24**		
	Psychopathy	0.02		
	Narcissism	0.05		
2	Competition	–0.01	0.27**	0.01
	Verification	−0.07*		
**Study 3 (*N* = 711): honest IM intentions**				
1	Honesty-humility	0.00	0.00	0.00
	Machiavellianism	0.01		
	Psychopathy	–0.02		
	Narcissism	0.04		
2	Competition	0.10**	0.02*	0.02**
	Verification	0.08*		

*p < 0.05, **p < 0.01.

### Discussion

As predicted, we found that high competition led to stronger faking intentions compared to low competition. In addition, we found a significant negative relation between Honesty-humility and faking intentions. These results are in line with previous research (Law et al., 2016; Buehl and Melchers, 2017; Roulin and Bourdage, 2017; Bourdage et al., 2018; Ho et al., 2019) and with existing faking models (Snell et al., 1999; Levashina and Campion, 2006; Roulin et al., 2016). However, contrary to our expectations, there was no significant difference in participants’ faking intentions between the verification warning condition and the no warning condition. A possible explanation why the verification warning had no impact could be that psychology students in Germany usually apply for many Master’s programs so that they might have perceived the verification warning as unrealistic given the vast numbers of applications in each program that make it challenging to really verify answers from specific applicants. Alternatively, another possibility could be that even if we gave no information about any verification in the no warning condition, we cannot rule out that participants still expected a verification of their answers in this condition. Maybe this is also the reason why the effect size from the manipulation check for perceived competition was much bigger than the effect size for perceived verification. Because of the non-significant effect of verification and the small significant effect of competition, it is also not surprising that these variables could not explain additional variance in faking intentions beyond the dispositional variables.

We also found non-significant correlations between Extraversion, Conscientiousness, Neuroticism, and Agreeableness on the one side and faking intentions on the other side. Even though we had expected some significant correlations, it should be mentioned that relationships for the Big Five in previous studies were also not always significant and the highest correlations were only medium-sized (cf. the results reviewed by Melchers et al., 2020) so that the present results are not completely surprising.

## Study 2

A limitation of Study 1 is that our sample only consisted of students. Furthermore, given the limited support for situational antecedents of faking intentions, it seemed advisable to investigate the assumed effects in another sample. Therefore, we conducted Study 2 with a sample of working participants to examine the generalizability of these results.

### Methods

#### Participants

Hundred-twenty-four German-speaking working individuals completed our online-survey. After excluding participants who answered more than one content-related question (see below) incorrectly, 114 individuals (83 females, 30 males, 1 no specification) were left for analyses. Participants’ mean age was 41.29 (*SD* = 10.29). 53.5% of them worked full time, 41.2% part-time and 5.3% were not currently working (e.g., because of parental leave). Their average interview experience was 7.66 interviews (*SD* = 5.97).

The participants were recruited through personal contacts. Requirements for participation were a job and at least one previous selection interview. All of them took part voluntary.

#### Procedure

The design and procedure of Study 2 were the same as in Study 1 with the exception that the vignettes were adapted to the working sample. Specifically, the vignettes now asked participants to imagine that they wanted an occupational change because too much routine had crept into their daily work routine. Accordingly, participants should imagine that they had applied for a new job and were invited to a selection interview.

#### Vignettes

To manipulate competition, we varied the description of the labor market situation. In the high competition condition, the economic situation of their sector was bad, companies were downsizing, and did not look for new employees. Even better qualified applicants they knew were looking for a job for a very long time. Additionally, participants were told that the selection ratio was very low (5 jobs for 70 applicants). In the low competition condition, we told participants that there were many vacant positions, even less qualified applicants they knew got attractive jobs, and the selection ratio was high (7 jobs for 10 applicants).

In the verification warning condition, it was said that applicants had to provide contact data of their supervisors and some coworkers to verify their interview answers. We additionally told them that these references were actually contacted and some previous applicants were excluded because they had provided false information. In the no warning condition, there was no cue that the organization would verify interviewees’ answers.

#### Measures

##### Personality

We used the same scales as in Study 1 to measure the Big Five and Honesty-humility. Coefficient alpha ranged from 0.63 to 0.79.

##### Faking intentions

The intention to fake was also measured with the same scale as in Study 1 (alpha = 0.83).

##### Manipulation check

We adapted the items from Study 1 to the new context. For example, we measured perceived competition (e.g., “The competition for jobs is quite high.”) with two items (alpha = 0.91) and verification with three items (e.g., “The company will verify my information from the interview.”; alpha = 0.72).

##### Content-related questions

Participants had to answer five content-related items, which were adapted to the new vignettes (e.g., “I have received an invitation for a selection interview.”) to be *true* or *not true*.

### Results

Table 4 shows correlations among all variables. To check whether the experimental manipulation worked as intended, we conducted two separate *t*-tests with the independent variables competition and verification warning and the corresponding dependent variables perceived competition and perceived verification. We found significantly higher values for perceived competition in the high competition condition (*M* = 4.72, *SD* = 0.69) than in the low competition condition (*M* = 1.88, *SD* = 0.81), *t*(102.66) = 20.09, *p* < 0.01, *d* = 3.78. In addition, we found significantly higher values for perceived verification in the verification warning condition (*M* = 4.40, *SD* = 0.48) than in the no warning condition (*M* = 2.68, *SD* = 0.87), *t*(85.00) = 12.91, *p* < 0.01, *d* = 2.45.

**TABLE 4:** Correlations, means, and standard deviations among all study variables in Study 2.

Variable	*M*	*SD*	1	2	3	4	5	6	7	8	9	10	11
(1) Sex	0.73	0.44	−										
(2) Age	41.29	10.28	–0.03	−									
(3) Extraversion	3.49	0.83	0.13	–0.07	0.79								
(4) Conscientiousness	3.86	0.68	0.17	–0.02	0.11	0.68							
(5) Neuroticism	2.87	0.84	0.08	–0.04	−0.27**	–0.10	0.67						
(6) Agreeableness	3.35	0.78	0.26**	0.17	0.05	0.17	0.00	0.66					
(7) Openness	3.77	0.69	0.15	0.05	0.19	0.27**	0.11	0.05	0.64				
(8) Honesty-humility	3.88	0.54	0.29**	0.18	–0.04	0.21*	0.02	0.32**	0.15	0.63			
(9) Competition	0.54	0.50	0.20*	0.04	0.00	0.06	0.10	0.08	0.02	0.03	−		
(10) Verification warning	0.51	0.50	–0.08	0.03	–0.1	0.15	–0.10	0.12	0.07	0.09	0.18	−	
(11) Faking intentions	2.36	0.59	–0.17	−0.23*	–0.05	−0.25**	0.06	–0.17	–0.12	−0.47**	–0.08	0.22*	0.83

N = 114. Sex was coded as 0 = male, 1 = female. Values in the diagonal show coefficient alpha for the different variables. *p < 0.05, **p < 0.01.

To evaluate the impact of our situational variables on faking intentions, we conducted a 2 × 2 ANOVA with the independent variables competition and verification warning. In line with Hypothesis 3, the verification warning lowered faking intentions compared to the no warning condition, *F*(3,110) = 4.98, *p* = 0.03, η^2^ = 0.043. However, in contrast to Hypothesis 1 – and also in contrast to Study 1 – the main effect for competition was non-significant, *F*(3,110) = 0.24, *p* = 0.62, η^2^ = 0.002. In addition, the Competition × Verification interaction was also non-significant, *F*(3,110) = 0.41, *p* = 0.53, η^2^ = 0.004 (cf. Table 2 for means and *SD*s).

To examine the relations between the dispositional variables and faking intentions, we conducted correlation analyses. In line with Hypotheses 4 and 6, we found significant negative correlations between Conscientiousness, *r* = −0.25, *p* = 0.01, and Honesty-humility, *r* = −0.47, *p* < 0.01, on the one hand and faking intentions on the other hand. In contrast to Hypothesis 5, the correlation between Neuroticism, *r* = 0.06, *p* = 0.55, and the intention to fake was non-significant. Concerning Research Questions 1 and 2, corresponding correlations for Extraversion, *r* = −0.05, *p* = 0.64, and Agreeableness, *r* = −0.17, *p* = 0.07, were also non-significant. For the sake of completeness, we also determined the correlation for Openness, which was also non-significant, *r* = −0.12, *p* = 0.20.

Finally, we conducted hierarchical multiple regressions (cf. Table 3) to examine whether the situational variables can explain variance in faking intentions beyond the dispositional variables. Again, we added Extraversion, Conscientiousness, Neuroticism, Agreeableness as well as Honesty-humility in Step 1 and competition and verification warning in Step 2. Step 1 explained a significant amount of variance (*R*^2^ = 0.25). Again, Honesty-humility was the only significant predictor. In contrast to Hypothesis 8 but in line with Study 1, there was no significant change in the amount of additional variance in Step 2 and the individual predictors competition and verification warning were both non-significant.

In addition to the tests of our hypotheses and research questions, we also conducted some additional explorative analyses, because faking intentions seemed higher in Study 1 (overall *M* = 2.83, *SD* = 0.59) than in Study 2 (*M* = 2.36, *SD* = 0.59). Specifically, we conducted a *t*-test with Study 1 vs. 2 as the independent variable and faking intentions as the dependent variable. This test confirmed that psychology students in Study 1 indeed had higher faking intentions than working individuals in Study 2, *t*(208) = 5.69, *p* < 0.01, *d* = 0.80.

### Discussion

In contrast to Study 1, we found that a verification warning led to lower faking intentions compared to no warning but that competition did not affect faking intentions. We found this result although the effect size from the manipulation check for perceived competition was again larger than the effect size for perceived verification. However, we replicated the negative correlations between Honesty-humility and faking intentions from Study 1 and found an additional significant correlation between Conscientiousness and faking intentions. Finally, we also replicated the Study 1 result that situational predictors did not explain incremental variance in faking intentions beyond the effects of personality, which were again largely due to Honesty-humility.

Taken together, Studies 1 and 2 suggest that personality variables (albeit not the Big Five) are more consistently related to faking intentions than situational variables. Furthermore, even though we initially aimed at designing Study 2 so that it should be parallel to Study 1 – with the exception that a working sample was used – the results did not turn out parallel. Thus, whereas competition was the only significant situational antecedent in Study 1, verification was the only significant situational antecedent in Study 2. In addition, our additional analyses also suggest that the student sample in Study 1 was more likely to fake in an interview than the working sample in Study 2. However, it is unclear whether this is due to the different vignettes or due to the different samples.

## Study 3

To rule out that the different results between Study 1 and 2 were due to the different vignettes we conducted Study 3 with identical vignettes for all participants and investigated participant background (non-student vs. student) as an additional quasi-experimental variable. Furthermore, given the relatively low values for participants faking intentions we also wanted to get more detailed insight into why they decided to use (or not to use) faking. Therefore, we also examined the reasons for the intention to show (vs. not to show) faking. We also excluded the Big Five because of the small and/or non-significant correlations and added the Dark Triad traits as personality predictors. Additionally, we included measures of honest IM in addition to measures of faking. Finally, a potential limitation of our first two studies is that we only considered additive effects of the situational and dispositional variables but no potential interactions between these two types of variables. Different theoretical models propose such interactions, but given the sample size in Studies 1 and 2, the test of interaction effects would make little sense because of the limited statistical power. However, the collection of a larger data set in Study 3 allowed us to test potential interaction effects with more power.

### Methods

#### Participants

Eight-hundred-twenty-seven participants completed the online survey for Study 3. However, we excluded 57 individuals because they answered more than one content-related question incorrectly. Furthermore, of the remaining individuals 59 answered the attention check incorrectly (see below). Thus, the final sample consisted of 711 participants (466 females, 240 males, 5 no specification) of whom 358 (50.4%) were students and 353 (49.6%) were non-students. Participants’ mean age was 30.77 years (*SD* = 10.64).

The participants were recruited via an online platform and social media (LinkedIn, Xing). Additionally, we used flyers to gain more attention. The students could take part in this study to partially fulfill a course requirement.

#### Procedure

We used a 2 × 2 × 2 between-subjects design (non-students vs. students, verification warning vs. no warning, and high vs. low competition) that was again administered via an online survey. Participants were first asked about demographic information (age, sex, and student status). Then, one of the four vignettes was randomly presented. Participants should imagine that they did not have a job but that they were invited to a selection interview by an attractive organization. After reading the vignette, participants had to answer several manipulation check questions. Then, they were asked to what extent they would use faking and honest IM and about their reasons for the intention to show faking or not. Finally, they had to complete the personality scales.

#### Vignettes

We varied the description of the labor market similar to Study 2 to manipulate competition. In the high competition condition, the economic situation of the industry was described as difficult (downsizing), many equally or better qualified individuals were searching for a new job, and the selection ratio was low (10 applicants for 1 job). In the low competition condition, the economic situation of the industry was described as good (many new jobs), even less qualified applicants had no problem to find a job, and the selection ratio was high (10 applicants for 9 jobs).

To manipulate verification, participants in the verification warning condition were told to imagine that they had to specify three references, the organization would contact these references to verify their interview answers, and applicants who provided false information would be excluded from this and future application processes. In the no warning condition there was no information about any attempts to verify interviewees’ answers.

#### Measures

##### Honesty-humility

We measured Honesty-humility with the same 10-item scale used in Studies 1 and 2 (alpha = 0.65).

##### Dark Triad

The Dark Triad was measured with the German version from Küfner et al. (2015) of the Naughty Nine by Jonason and Webster (2010). This 9-item measure consists of three scales: Machiavellianism (e.g., “I tend to manipulate others to get my way.”; alpha = 0.72), Narcissism (e.g., “I tend to want others to admire me.”; alpha = 0.83), and Psychopathy (e.g., “I tend to be callous or insensitive.”; alpha = 0.55).

##### Faking and honest IM intentions

We measured faking intentions with a 16-item version (e.g., “I would distort my answers based on the comments or reactions of the interviewer.”; alpha = 0.87) of Levashina and Campion’s (2007) faking scale developed by Bourdage et al. (2018). In comparison to the Ingold et al. (2015) scale, this scale is a somewhat more comprehensive measure and contains a few more items from the four subfacets of interview faking. Thereby, it represents a measure that is also more parallel to the Bourdage et al. (2018) measure of honest IM. Additionally, we measured honest IM with a 12-item scale (alpha = 0.75) from Bourdage et al. (2018), which contains the three subfacets of honest IM (e.g., “I would make sure to let the interviewer know about my job credentials.”). All items were adapted to the hypothetical scenario and had to be answered on 5-point scales from 1 = *to no extend* to 5 = *to a very great extent*.

##### Reasons for the intention to show vs. not to show faking

To examine the reasons to show or not to show faking, we asked participants “How would you present yourself in relation to the situation described above?”. This item had to be answered on a 6-point scale from 1 = *I would try to present myself as honestly as possible* to 6 = *I would try to present myself better than I really am*. If respondents chose an answer between 1 and 3, they were presented with the open-ended question: “Why would you NOT try to present yourself better than you really are?”. And if they chose an answer between 4 and 6, they were presented with the open-ended question: “Why would you try to present yourself better than you really are?”. It was possible for participants to provide several reasons for their intention.

##### Manipulation check

We measured perceived competition (alpha = 0.90) and perceived verification (alpha = 0.76) with two items each. All items were taken from Study 2. We only shortened the verification scale by one item and adapted the competition items slightly. The items had to be rated on a 7-point scale from 1 = *I do not agree at all* to 7 = *I fully agree*.

##### Content-related questions

To ensure that the participants read their vignettes carefully, they had to answer five content-related items as *true* or *not true*. Two items were taken from Study 2 and the other three items were developed for this study.

##### Attention check

We distributed three different attention check questions (e.g., “I do not read the questions of this survey.”) across the whole questionnaire, to ensure that participants attentively read the items and the instructions of the survey. Two items had to be rated on a 5-point scale from 1 = *I do not agree at all*/*to no extend* to 5 = *I fully agree*/*to a very great extent*. The third attention check item instructed participants not to answer this item if they had read it. This item had the response options *yes* and *no*.

### Results

Correlations among all study variables are shown in Table 5. To determine whether the experimental manipulation worked as intended, we again conducted two separate *t*-tests. The high competition condition (*M* = 6.17, *SD* = 1.05) led to higher perceived competition than the low competition condition (*M* = 2.19, *SD* = 1.22), *t*(674.96) = 46.29, *p* < 0.01, *d* = 3.48. Additionally, the verification warning condition (*M* = 6.35, *SD* = 1.19) led to higher perceived verification than the no warning condition (*M* = 4.14, *SD* = 1.90), *t*(545.08) = 18.31, *p* < 0.01, *d* = 1.40.

**TABLE 5:** Correlations, means, and standard deviations among all study variables in Study 3.

Variable	*M*	*SD*	1	2	3	4	5	6	7	8	9	10	11
(1) Sex	0.66	0.47	−										
(2) Age	30.77	10.64	−0.30**	−									
(3) Student	0.50	0.50	0.24**	−0.61**	−								
(4) Honesty-humility	3.68	0.59	0.09*	0.12**	–0.05	0.65							
(5) Machiavellianism	2.11	0.88	–0.06	−0.10**	0.08*	−0.49**	0.72						
(6) Psychopathy	1.57	0.69	−0.25**	0.02	0.00	−0.30**	0.36**	0.55					
(7) Narcissism	2.76	1.01	0.06	−0.18**	0.13**	−0.47**	0.38**	0.15**	0.83				
(8) Competition	0.52	0.50	–0.03	0.05	–0.07	–0.03	–0.02	0.00	0.03	−			
(9) Verification warning	0.53	0.50	0.04	0.03	–0.02	0.02	0.04	–0.05	–0.04	0.01	−		
(10) Honest IM	3.79	0.50	0.07	0.00	0.03	–0.01	0.01	–0.02	0.04	0.10**	0.08*	0.75	
(11) Faking intentions	2.20	0.58	0.04	−0.20**	0.19**	−0.45**	0.42**	0.21**	0.29**	0.00	–0.07	0.17**	0.87

N = 711. Sex was coded as 0 = male, 1 = female; student was coded as 0 = non-students, 1 = students; IM = impression management. Values in the diagonal show coefficient alpha for the different variables. *p < 0.05, **p < 0.01.

Next, we conducted a 2 × 2 × 2 ANOVA with faking intentions as the dependent variable and the independent variables competition, verification warning, and student status. The results revealed a significant main effect of student status, *F*(7,703) = 24.76, *p* < 0.001, η^2^ = 0.034, which reflects that students had higher faking intention than non-students. In contrast to Hypotheses 1 and 3, the main effect for competition, *F*(7,703) = 0.04, *p* = 0.84, η^2^ = 0.000, and for the verification warning were both non-significant, *F*(7,703) = 3.06, *p* = 0.08, η^2^ = 0.004. Furthermore, none of the interactions turned out to be significant, all *F*s < 0.13 (cf. Table 2 for means and *SD*s).

To evaluate effects on honest IM intentions, we conducted another 2 × 2 × 2 ANOVA with the independent variables competition, verification warning, and student status. In line with Hypothesis 2, this ANOVA confirmed that high competition led to stronger honest IM intentions than low competition, *F*(7,703) = 7.67, *p* < 0.01, η^2^ = 0.011. We also found that the verification warning led to more honest IM intentions than the no warning condition, *F*(7,703) = 4.17, *p* = 0.04, η^2^ = 0.006. The main effect for student status and all interactions were non-significant, all *F*s < 1.08 (cf. Table 2 for descriptive information).

Next, we conducted correlation analyses to examine the relation for the dispositional variables. In line with Hypotheses 6 and 7, there was a significant negative correlation between faking intentions and Honesty-humility, *r* = −0.45, *p* < 0.01, and significant positive correlations between faking intentions and Machiavellianism, *r* = 0.42, *p* < 0.01, Psychopathy, *r* = 0.21, *p* < 0.01, and Narcissism, *r* = 0.29, *p* < 0.01.

To answer Research Questions 3 and 4, we also conducted correlation analyses with the dispositional variables and honest IM intentions. None of these correlations was significant and the corresponding *r*s ranged from −0.02 to 0.04.

Next, hierarchical multiple regressions (Table 3) were conducted to evaluate whether the situational variables can explain additional variance in faking intentions beyond the dispositional variables. As in the previous studies, the personality variables from Step 1 explained a significant amount of variance (*R*^2^ = 0.26). Honesty-humility and Machiavellianism were significant predictors. In contrast to Hypothesis 8 but in line with the previous studies, there was no significant change in the amount of explained variance from Step 1 to Step 2. Nevertheless, verification was a significant situational predictor.

In addition, we also conducted a hierarchical multiple regression (Table 3) to examine whether the situational variables can explain additional variance in honest IM intentions beyond the dispositional variables. The personality variables in Step 1 did not explain a significant amount of variance but there was a significant change from Step 1 to Step 2, Δ*R*^2^ = 0.02, *p* < 0.01. The situational predictors competition and verification were both significant.

Next, we conducted several hierarchical multiple regressions to test Hypothesis 9 and to examine whether there were significant interactions between situational and dispositional variables in explaining faking intentions. For each interaction between a situational and a dispositional variable, we conducted a separate regression analysis. In Step 1 of each analysis, we added competition or verification warning and one of the dispositional variables into the regression. In Step 2, we added the corresponding interaction between these two variables. Hardly any support for the predicted interaction effects was found because only the Competition × Machiavellianism interaction was significant, β = −0.11, *p* = 0.02. Furthermore, Step 2 of the regression with competition and Machiavellianism only explained 0.6% of additional variance beyond Step 1.

**FIGURE 1:**
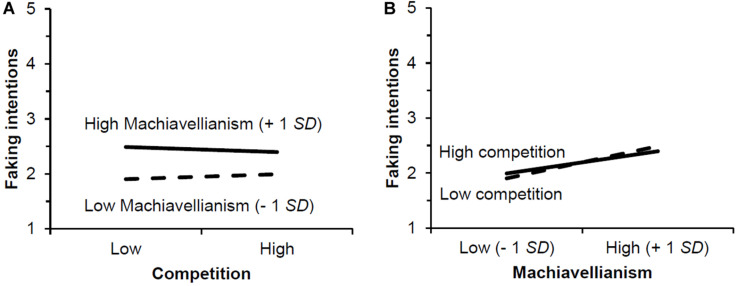
Graphical illustration of the interaction between competition and Machiavellianism. Panel **(A)** shows simple slopes for Machiavellianism as the moderator and **(B)** shows simple slopes for competition as the moderator.

Concerning the Competition × Machiavellianism interaction, a test of the simple slopes with Machiavellianism as the moderator revealed that the relationship between competition and faking intentions was negative but non-significant for high (+1 *SD*, *b* = −0.09, *p* = 0.10) and positive but also non-significant for low (−1 *SD*, *b* = 0.09, *p* = 0.11) Machiavellianism (cf. Figure 1A). In contrast to this, when we calculated simple slopes with competition as the moderator of the relationship between Machiavellianism and faking intentions, the relationship was significant for high (+1 *SD*, *b* = 0.23, *p* < 0.01) and for low (−1 *SD*, *b* = 0.33, *p* < 0.01) competition (cf. Figure 1B). For the sake of completeness, we also conducted hierarchical multiple regressions for honest IM intentions but found no significant interactions between situational and dispositional variables.

Finally, we considered participants’ answers to the question how they would present themselves in the described situation and we analyzed the reasons for the intention to show vs. not to show faking. To do so, we clustered participants’ answers for the corresponding questions. If an answer contained more than one reason, we split the answer so that we could distinguish between the different reasons. The mean for the rating item was 2.20 (*SD* = 1.16) which was much closer to the honest end of the scale than to the dishonest end. In line with this, the 110 participants whose answers were from the dishonest half of the scale (4–6) provided only 169 reasons why they would try to present themselves more positively than they are. In contrast, the 601 participants whose answers were from the honest half of the scale (1–3) provided 1,121 reasons why they would not try to present themselves more positively than they are. The most frequently mentioned reasons for the intention to show faking were that faking increases chances to get the job (approximately 17%), that others present themselves better as well (12%), and competition (9%). In contrast, the most frequently mentioned reasons to show no faking were that lying will be revealed and/or has negative consequences (36%), ethical and moral reasons (13%), and that participants want to enable a good person-organization and/or person-job fit. Further reasons for the intention to show faking or to show no faking are shown in Table 6.

**TABLE 6:** Reasons for the intention to show vs. not to show faking.

Reason	Frequency	Percentage
**Reasons for the intention to show faking (*n* = 110):**		
Increases chances to get the job	29	17.16
Others present themselves better as well	21	12.43
Competition	16	9.47
You have to sell yourself well	14	8.28
I can still acquire the promised skills/abilities	9	5.33
Unemployed	7	4.14
Faking is expected	7	4.14
Honesty harms	7	4.14
Faking as an important part of the application	5	2.96
Enhancing of one’s own attractiveness	4	2.37
Cannot be verified	4	2.37
Organizations present themselves better as well	3	1.78
To attain a better salary	3	1.78
Attractiveness of the job	3	1.78
I’m better than I think	2	1.18
Compensation of one’s own deficits	2	1.18
Is recommended by guidebooks	2	1.18
Others	31	17.75
Total	169	100
**Reasons for the intention to show no faking (*n* = 601):**		
Lying will be revealed and/or has negative consequences	403	35.95
…in or directly after the interview	119	29.53
…in the long-term during work	142	35.24
…not specified	142	35.24
Ethical and moral reasons	146	13.02
Person-organization fit/person-job fit	113	10.08
Concerns about creating too high performance expectations	97	8.65
Honesty pays off	86	7.67
Faking is not necessary	79	7.04
Honesty as the basis for a good relationship	57	5.08
Insufficient belief in one’s own abilities to lie	31	2.77
Lying is exhausting and stressful	27	2.41
Would like to convince with own qualities	24	2.14
Enabling a good selection decision	9	0.80
Lying is unlikeable	7	0.62
Testing of the employer through honesty	3	0.27
Others	39	3.48
Total	1121	100

### Discussion

In contrast to Studies 1 and 2 neither competition nor verification affected faking intentions. Furthermore, even though we had a larger sample size and therefore more power than in Studies 1 and 2, the situational variables could again not explain a significant amount of variance beyond the dispositional variables. The small impact of the situational variables is also reflected in the reasons for the intention to show faking that participants provided to the open-ended question. Only about 9% of these reasons included competition. Similarly, concerning the reasons for their intention to show no faking, the non-significant effect for verification also does not seem surprising, either. About 36% of the reasons included the fear that lies would be discovered and/or would lead to negative consequences but only about a third of these answers included the fear of being caught directly in or after the interview. Given that the verification warning was aimed at being caught directly after the interview, most of the reasons mentioned by the participants were therefore unrelated to our manipulation. However, as hypothesized, we found a significant negative correlation between Honesty-humility and also significant positive correlations between all facets of the Dark Triad and faking intentions. Furthermore, concerning the correlations between the Dard Triad and faking intentions, Machiavellianism had the highest and Psychopathy the lowest correlations. However, as a caveat we would like to note that our Psychopathy scale had a relatively low internal consistency. Finally, we confirmed the impression from Studies 1 and 2 that students report larger faking intentions than non-students.

In addition to the main effects of situational and dispositional variables, we also examined interactions between both types of variables in Study 3. In contrast to different faking theories, we found hardly any support for the predicted interaction effects. Specifically, the only significant interaction occurred between Machiavellianism and competition and the direction of the interaction was at variance with predictions from the model by Roulin et al. (2016), which predicts a stronger relationship between competition and faking intensions for people who are high on Machiavellianism. In contrast to this, the direction of the interaction was in agreement with the model from McFarland and Ryan (2000), which predicts that personality is more strongly related to faking intentions in the absence of strong situational variables. However, this interaction could only explain 0.6% of the additional variance in faking intentions. Together with the absence of other significant interactions, this suggests that there was hardly any support for the predicted moderator effects.

Concerning honest IM, we confirmed our expectation that high competition led to more honest IM intentions compared to low competition. Additionally, the verification warning also led to larger honest IM intentions compared to the no warning condition. Furthermore, the situational variables also explained additional variance beyond the dispositional variables. However, in contrast to faking intentions, we found that the dispositional variables Honesty-humility and the Dark Triad were unrelated to honest IM intentions. Thus, it is noticeable that competition and verification warnings had a positive effect on honest IM intentions but not on faking intentions. In contrast, the Dark Triad and Honesty-humility were mainly related to faking intentions but not to honest IM intentions. We will discuss possible reasons for this pattern in more detail below in the Main Discussion. However, altogether, these results support the conclusions by Bourdage et al. (2018) that faking and honest IM have different antecedents.

## Main Discussion

The present studies belong to the few studies that investigated the impact of situational variables on faking intentions in selection interviews. Furthermore, by also collecting qualitative answers in addition to quantitative information, our research helps to gain a better understanding of faking and honest IM intentions in selection interviews. Based on this, the present results make several relevant contributions to interview faking research.

### Situational Variables

All three studies revealed that competition has only little or no impact on faking intentions. We only found slightly higher faking intentions in a high competition condition compared to a low competition condition in Study 1. However, even there, the effect size for this difference was rather small. These results are only partially in line with the dynamic model of applicant faking by Roulin et al. (2016), which assumes that high competition leads to a higher motivation to fake and, therefore, affects actual faking. Nevertheless, other studies also found small effects (Ho et al., 2019) or no effect (Buehl and Melchers, 2018). In the present studies, a possible reason could be that not every applicant could expect to get a job or a place for a Master’s program in the low competition conditions. Thus, although the manipulation check worked as intended, it is possible that the low competition manipulation was still sufficient to trigger faking intentions in the same way as the high competition manipulation. Another possible reason for the present results can be found in the qualitative results in Study 3. There, it turned out that competition was no dominant reason to use faking which can be seen by the fact that only about 9% of the answers concerning intentions to show faking included competition. More important reasons for the intention to show faking were to generally increase the chances to get the job (17%) and that others present themselves better as well (12%). And finally, it might also be that the hypothetical nature of the selection situation in our vignettes (as well as in all the other previous studies that considered the impact of competition on interview faking) attenuates the actual effects that competition would have in a real world high-stakes selection situation.

In addition to competition, we also examined the effect of warning interviewees that information from their answers will be verified. As with competition, all three studies found that verification warnings had little or no impact on faking intentions. These results provide only limited support for Levashina and Campion’s (2006) faking model and the dynamic model of faking by Roulin et al. (2016). Both models assume that a higher probability of getting caught should reduce faking. Our results are also only partially in line with the only study that examined the effects of warnings on faking behavior in an interview context by Law et al. (2016). However, in contrast to our studies, Law et al. (2016) told their participants that they would be asked questions in the interview that are suitable to identify faking. In our studies, we told participants that their answers would be verified by means of references. As a result, participants may have found our verification warning more controllable, for example, by only providing contact details for references for which it seems likely that these also provide positive or positively biased information (Leising et al., 2010). Therefore, it may be that the warning used by Law et al. (2016) may have had a greater impact on intentions to use faking. Another possible explanation for our results can be found in the qualitative results in Study 3. The most frequently mentioned reason for the intention to show no faking was indeed that lying would be revealed and/or would have negative consequences (36%) but only about a third of these reasons included the fear of being caught directly in or after the interview. In contrast, many of these reasons included the fear that lies will be revealed in the long-term at work or were not specified. Therefore, it seems understandable why the verification manipulation had hardly any effect on the intention to show faking because most reasons were unaffected by our manipulation. Nevertheless, because of the hypothetical nature of the selection scenario in our studies, the same caveat as for competition is relevant here with regard to the generalizability of the present results to high-stakes field settings.

Apart from this, we also examined the impact of competition and verification warnings on honest IM in Study 3. As expected and in contrast to the results for faking, we found higher honest IM intentions in a high competition condition compared to a low competition condition. This result extends findings by Bourdage et al. (2018) that honest IM and faking have different antecedents. However, it should also be noted that this effect was only small. Additionally, we found higher intentions to use honest IM in a verification warning condition compared to a no warning condition. This result was not hypothesized and we were surprised that a warning about verification encouraged participants to use more honest IM. However, again the corresponding effect size was very small.

### Dispositional Variables

With regard to the relationship between dispositional variables and faking intentions, our results replicate and extend earlier findings. First, the Big Five had only limited impact on faking intentions so that only Conscientiousness had a small to medium negative relation with faking intentions in one study. These results are in contrast to the faking model by Levashina and Campion (2006) but in line with the overall pattern from previous studies in which correlations between the Big Five and faking were usually in the small to moderate range (Buehl and Melchers, 2017; Roulin and Bourdage, 2017; Bourdage et al., 2018; Melchers et al., 2020). In contrast to the Big Five, we found consistent and strong negative relations between Honesty-humility and faking intentions across all three studies. These results are in line with Levashina and Campion’s (2006) model and with previous studies (Law et al., 2016; Buehl and Melchers, 2017; Roulin and Bourdage, 2017; Bourdage et al., 2018; Ho et al., 2019). However, in this regard it should be noted that there is considerable conceptual overlap between the Honesty-humility items (e.g., “I wouldn’t use flattery to get a raise or promotion at work, even if I thought I would succeed.”) and items to measure faking intentions that focus on ingratiation, or (slight or extensive) image creation. This could also be a reason why we found so strong negative correlations between these two variables. Additionally, we also found consistent positive correlations between all facets of the Dark Triad and faking intentions in Study 3, which is also in line with Levashina and Campion (2006) and with similar results found in other studies (Roulin and Krings, 2016; Roulin and Bourdage, 2017). Thus, besides Honesty-humility, the Dark Triad seem to be important predictors for faking intentions, too. Furthermore, similar to Honesty-humility the Dark Triad were more important predictors than the Big Five.

In addition to faking intentions, Study 3 also extended the sparse research on honest IM. In that study, we also found that Honesty-humility and all the Dark Triad dimensions were unrelated to honest IM intentions which replicates previous findings by Roulin and Bourdage (2017). Furthermore, our results extend earlier findings by Bourdage et al. (2018) that honest IM and faking have different antecedents.

As another contribution, our study also provides initial evidence concerning the relative importance of situational vs. dispositional antecedents of faking. Even though the direct comparison between these two kinds of antecedents might not be entirely fair (given that only situational factors were manipulated experimentally) the results across our three studies suggest that dispositional variables might be more relevant for faking intentions than situational variables. As the situational variables competition and verification warning did not explain incremental variance beyond the dispositional variables, dispositional variables were the primary driver that influenced faking intentions in our studies.

Our finding that honest IM seems more susceptible to situational factors than faking whereas faking seems more susceptible to dispositional factors is also noticeable. A possible reason for this could be that most of the participants were reluctant in general with regard to using faking behavior as can be seen by the low means for faking intentions in all three studies and by the answers to the open-ended question in Study 3. Thus – at least in these hypothetical situations – it seems as if only individuals with certain personality profiles think about using dishonest means to improve their chances in selection interviews but they seem to do this independently of the specific situation. In contrast to this, people in general seem to have less reservations to increase their use of honest IM in situations where this seems necessary to them but in which they still do not have to deviate from the truth.

Finally, we tested interaction effects between situational and dispositional variables that were predicted by several faking models. However, we only found an interaction between Machiavellianism and competition, the interaction accounted for rather little variance, and the direction of this interaction was in contrast to the faking model by Roulin et al. (2016), which would predict a stronger relationship between competition and faking intensions for people who are high on Machiavellianism. The other interactions between verification warnings or competition on one hand and Honesty-humility, Narcissism, or Psychopathy on the other hand were not significant. Thus, in contrast to several faking models but in line with results concerning personality testing by McFarland and Ryan (2006), little evidence for moderator effects of situational variables such as a warning against faking were found.

### Student Status

As another relevant contribution to interview faking research, we found higher faking intentions for students compared to non-students in Study 3. We also found higher faking intentions for the student sample in Study 1 compared with the working sample in Study 2. Similarly, a review of previous research suggests a negative relationship between age and faking in selection interviews (Melchers et al., 2020) even though no previous study in the interview domain formally tested the relationship between age and faking (in that review, corresponding information for the correlation with age was extracted from correlation tables from primary studies that were designed to investigate other variables). Furthermore, results from research in the domain of unproctored internet testing also suggest that recent university graduates are more prone to show dishonest test responses in comparison to other groups of applicants (Lievens and Burke, 2011). Thus, it seems that students, university graduates, and/or younger applicants have a stronger tendency to show dishonest behavior during an application process. As a reason for this, Bourdage et al. (2018) suggested that younger applicants may try to compensate for their lack of qualifications by using faking. Moreover, most of them probably have limited work experience, which they could also try to compensate by faking.

### Practical Implications

Our results have several implications for personnel selection in organizations. First, the impact of information about a verification warning on faking intentions was negligible. Based on our results, we suggest not to inform applicants that information from their answers will be verified because this does hardly seem to prevent them from faking. Second, given the minimal impact of competition on faking intentions, it also seems that organizations do not have to worry too much that faking becomes more common in a highly competitive environment because it only had a small effect on faking intentions. Third, for organizations it seems to make more sense to try to identify individuals with a strong inclination to fake. Thus, organizations should try to gain more information concerning individual difference variables such as Machiavellianism, Psychopathy, Narcissism, or Honesty-humility because individuals with high scores on the traits from the Dark Triad or low scores for Honesty-humility tend to fake more in selection interviews. Furthermore, given that the same pattern of scores is also related to more deviant on-the-job behavior (e.g., O’Boyle et al., 2012; Pletzer et al., 2019), it seems advisable for organizations to seek for ways to prevent corresponding individuals from entering an organization in the first place. And finally, it could also be useful to take measures that make faking more difficult instead of trying to warn applicants not to fake. Specifically, previous research found that standardization reduces the influence of IM tactics on interviewer ratings (Barrick et al., 2009). Therefore, organizations could, for example, use higher degrees of standardization for their interviews to reduce the potential influence of faking on interview outcomes.

### Limitations and Future Research

Although, we conducted three studies to examine the impact of situational and dispositional variables on faking intentions, our research has some limitations. First, we only used hypothetical scenarios to manipulate competition and verification warnings. Therefore, we could only measure IM intentions and not actual IM behavior and these intentions were not measured in a high-stakes context. Thus, a caveat is necessary concerning the generalizability of the present results to field settings and future research is needed to evaluate the impact of information concerning verification warnings and of competition in real selection situations.

Second, our vignettes did not distinguish between different ways in which interviews might be designed and conducted. Specifically, previous research found that IM tactics have less impact on interview evaluations in highly standardized interviews than in less standardized interviews (Barrick et al., 2009) but it is unclear whether antecedents equally influence faking behavior in interviews that differ with regard to standardization. In addition, Bourdage et al. (2018) found hardly any effect of question type on faking when question type was assessed retrospectively on the basis of interviewees’ answers in a post-interview survey. However, the results by Barrick et al. (2009) suggest that the effects of question type and of interview standardization on faking should be evaluated with regard to their suitability to reduce potential effects of faking on interview performance (Buehl et al., 2019). Thus, future research is needed to evaluate this possibility.

Third, even though our third study provided new insight into antecedents of honest IM, more research is needed to identify additional situational antecedents of honest IM. Previous research found that honest IM is associated with question type, difficulty of the interview, interview duration, and type of interviewer (Bourdage et al., 2018). However, measures that allow to distinguish between honest vs. dishonest IM in interviews were only developed recently. Thus, in order to gain a better understanding of how honest IM and faking differ and what influences them, it is necessary to search for further antecedents.

Furthermore, the present results indicate that more research is needed concerning situational antecedents of faking in selection interviews. Especially knowledge on situational factors that help to reduce faking and that can be actively influenced by organizations is rare (Melchers et al., 2020). So far, only warnings in the form of an announcement that participants would be asked questions that are suitable to identify faking (Law et al., 2016) seem to have a beneficial effect. However, such a warning might have negative effects on applicant reaction variables.

Finally, with regard to avenues for future research, answers to the open-ended questions in Study 3 revealed possible starting points that have so far received too little or no attention. Relevant reasons to not use faking were that faking would lead to an impaired person-organization fit or person-job fit. This finding is in line with the self-presentation theory by Marcus (2009) that assumes that differences between the self-concept and the perceived ideal image negatively affect the valence of a job. We think it is also a matter of being able to exercise the job successfully and to feel comfortable. However, we are not aware of any study concerning the impact of interviewees’ perceived person-organization or person-job fit on faking. Another reason that has not been considered in research, yet, is the influence of performance expectations. Participants answered that presenting themselves better than they are would create too high performance expectations and, therefore, would not be an option for them. In addition, participants would also not use faking because they perceive honesty as important for a good relationship. On the other side, we also found reasons to show faking in selection interviews. Hereby, we also came across factors that seem relevant but that have been given too little or no attention by previous research. Specifically, participants argued that they would use faking because others present themselves better as well. Another interesting argument was that they could still acquire the promised skills/abilities. Altogether, we found some additional factors that could possibly influence faking in selection interviews and that should be considered by future research.

### Conclusion

Our results revealed that the situational variables competition and a warning about verification of given answers had hardly any effect on faking and honest IM intentions. Faking intentions were primary influenced by dispositional variables like Honesty-humility and the Dark Triad. Further research is needed to identify situational antecedents of faking and also to evaluate the generalizability of the present results in real interviews. In addition to our situational and dispositional variables, we found that students reported stronger faking intentions than non-students. Thus, younger applicant samples and recent university graduates seem to be more likely to fake in selection interviews.

## Data Availability Statement

All datasets generated for this study are included in the article/Supplementary Material.

## Ethics Statement

Ethical review and approval was not required for the study on human participants in accordance with the local legislation and institutional requirements. The participants provided their written informed consent to participate in this study.

## Author Contributions

A-KB and SW designed and conducted Studies 1 and 2. BB designed and conducted Study 3, analyzed the data from all three studies, and drafted the first version of manuscript. KM provided feedback to all the studies and to the analyses, and edited previous versions of the manuscript. All authors contributed to the article and approved the submitted version.

## Conflict of Interest

A-KB is employed by Carl-Zeiss AG, Oberkochen. The remaining authors declare that the research was conducted in the absence of any commercial or financial relationships that could be construed as a potential conflict of interest.

## Footnotes

1 In Germany, a Master’s degree is the usual degree that graduates need to be able to work as a psychologist afterward. Furthermore, usually there is relatively high competition for places in attractive Master’s programs because of the limited number of available places.
